# Markedly improving asymmetric oxidation of 1-(4-methoxyphenyl) ethanol with *Acetobacter* sp. CCTCC M209061 cells by adding deep eutectic solvent in a two-phase system

**DOI:** 10.1186/s12934-015-0407-1

**Published:** 2016-01-13

**Authors:** Ping Wei, Jing Liang, Jing Cheng, Min-Hua Zong, Wen-Yong Lou

**Affiliations:** State Key Laboratory of Pulp and Paper Engineering, South China University of Technology, Guangzhou, 510640 Guangdong People’s Republic of China; School of Chemistry and Chemical Engineering, South China University of Technology, Guangzhou, 510640 Guangdong People’s Republic of China; Lab of Applied Biocatalysis, College of Food Science and Engineering, South China University of Technology, Guangzhou, 510640 Guangdong People’s Republic of China

**Keywords:** Asymmetric oxidation, Immobilized *Acetobacter* sp. CCTCC M209061 cells, MOPE, Biphasic system, DES

## Abstract

**Background:**

Enantiopure (*S*)-1-(4-methoxyphenyl) ethanol {(*S*)-MOPE} can be employed as an important synthon for the synthesis of cycloalkyl [*b*] indoles with the treatment function for general allergic response. To date, the biocatalytic resolution of racemic MOPE through asymmetric oxidation in the biphasic system has remained largely unexplored. Additionally, deep eutectic solvents (DESs), as a new class of promising green solvents, have recently gained increasing attention in biocatalysis for their excellent properties and many successful examples in biocatalytic processes. In this study, the biocatalytic asymmetric oxidation of MOPE to get (*S*)-MOPE using *Acetobacter* sp. CCTCC M209061 cells was investigated in different two-phase systems, and adding DES in a biphasic system was also explored to further improve the reaction efficiency of the biocatalytic oxidation.

**Results:**

Of all the examined water-immiscible organic solvents and ionic liquids (ILs), 1-butyl-3-methylimidazolium hexafluorophoshpate ([C_4_MIM][PF_6_]) afforded the best results, and consequently was selected as the second phase of a two-phase system for the asymmetric oxidation of MOPE with immobilized *Acetobacter* sp. CCTCC M209061 cells. For the reaction performed in the [C_4_MIM][PF_6_]/buffer biphasic system, under the optimized conditions, the initial reaction rate, the maximum conversion and the residual substrate *e.e.* recorded 97.8 μmol/min, 50.5 and >99.9 % after 10 h reaction. Furthermore, adding the DES [ChCl][Gly] (10 %, v/v) to the aqueous phase, the efficiency of the biocatalytic oxidation was rose markedly. The optimal substrate concentration and the initial reaction rate were significantly increased to 80 mmol/L and 124.0 μmol/min, respectively, and the reaction time was shortened to 7 h with 51.3 % conversion. The immobilized cell still retained over 72 % of its initial activity after 9 batches of successive reuse in the [C_4_MIM][PF_6_]/[ChCl][Gly]-containing buffer system. Additionally, the efficient biocatalytic process was feasible up to a 500-mL preparative scale.

**Conclusion:**

The biocatalytic asymmetric oxidation of MOPE with *Acetobacter* sp. CCTCC M209061 cells was successfully conducted in the [C_4_MIM][PF_6_]-containing biphasic system with high conversion and enantioselectivity, and the reaction efficiency was further enhanced by adding [ChCl][Gly] to the reaction system. The efficient biocatalytic process was promising for the preparation of enantiopure (*S*)-MOPE.

## Background

Chiral alcohols are one kind of pivotal building blocks for synthesis of chiral pharmaceuticals, agrochemicals, flavors, fragrances and functional materials [1, 2]. Among them, enantiopure 1-(4-methoxyphenyl) ethanol (MOPE) is a key chiral building block. For example, (*S*)-1-(4-methoxyphenyl) ethanol {(*S*)-MOPE} can be employed for the synthesis of cycloalkyl [*b*] indoles which have the treatment function for general allergic response [3, 4]. And (*R*)-1-(4-methoxyphenyl) ethanol {(*R*)-MOPE} can be used for the preparation of chiral 3-aryl-3-substituted propanoic acids with anti-inflammatatory activity [5]. Currently, enantiopure chiral alcohols could be prepared mainly through chemical or biological approaches. Compared with chemical methods, biological methods have gained much attention owing to their mild reaction conditions, high enantioselectivity and being environmental friendly. Generally, whole microbial cells rather than isolated enzymes are used preferentially as the biocatalysts to avoid enzyme purification and coenzyme addition or the requirement for an additional system for coenzyme regeneration as well as the inactivation of the related enzymes by keeping them within the natural environments of cells. Additionally, utilization of immobilized microbial cells can not only facilitate separation of product, but also make biocatalysts recyclable, thus greatly simplifying the biocatalytic process and reducing the cost.

There are so far some reports on the biocatalytic synthesis of enantiomerically pure (*S*)-MOPE catalyzed by microbial cells, which focus on the biocatalytic asymmetric reduction of 4′-methoxyacetophenone (MOAP) [6–8]. To our knowledge, however, the biocatalytic resolution of racemic MOPE through whole cell-mediated asymmetric oxidation to obtain (*S*)-MOPE has remained largely unexplored. In our previous study [9], the whole cell of *Acetobacter* sp. CCTCC M209061 isolated from China kefir [10] was capable of catalyzing the asymmetric oxidation of MOPE in an aqueous monophasic system with above 98 % *e.e.* of (*S*)-MOPE. However, the optimal substrate concentration was only 30 mmol/L, limiting the industrial application of the biocatalytic process. In this case, it was found that the substrate and product had notably inhibitory and toxic effects on the microbial cells in the aqueous monophasic system, probably resulting in the low reaction efficiency. Generally, a biphasic system has been developed in order to solve the above-mentioned problems [11, 12], where an aqueous phase contains microbial cells and a water-immiscible organic solvent or ionic liquid phase acts as a reservoir for substrate and product. In many cases [13–16], use of a biphasic system especially containing more biocompatible ionic liquid was shown to be effective in lowering the inhibitory and toxic effects of substrate and product on microbial cells and thus increasing the concentration of reactant. Therefore, it was of great interest to investigate the biocatalytic asymmetric oxdiation of MOPE with *Acetobacter* sp. CCTCC M209061 cells in a two-phase system to boost the reaction efficiency.

Deep eutectic solvents (DESs), as a new generation of promising ionic liquid analogues composed of a quaternary ammonium salt and a metal salt or hydrogen bond donor [17, 18], have been applied in many respects [19–21]. Much attention has recently been paid to their applications in biocatalysis with successful results [22, 23], because of their nontoxic nature, good biodegradability and low cost. Up to now, few works have been published about the whole-cell biocatalysis in DES-containing systems [24–26]. In these cases, DESs were able to effectively prompt the biotransformations and manifested great potential for whole-cell biocatalytic process.

**Scheme 1:**
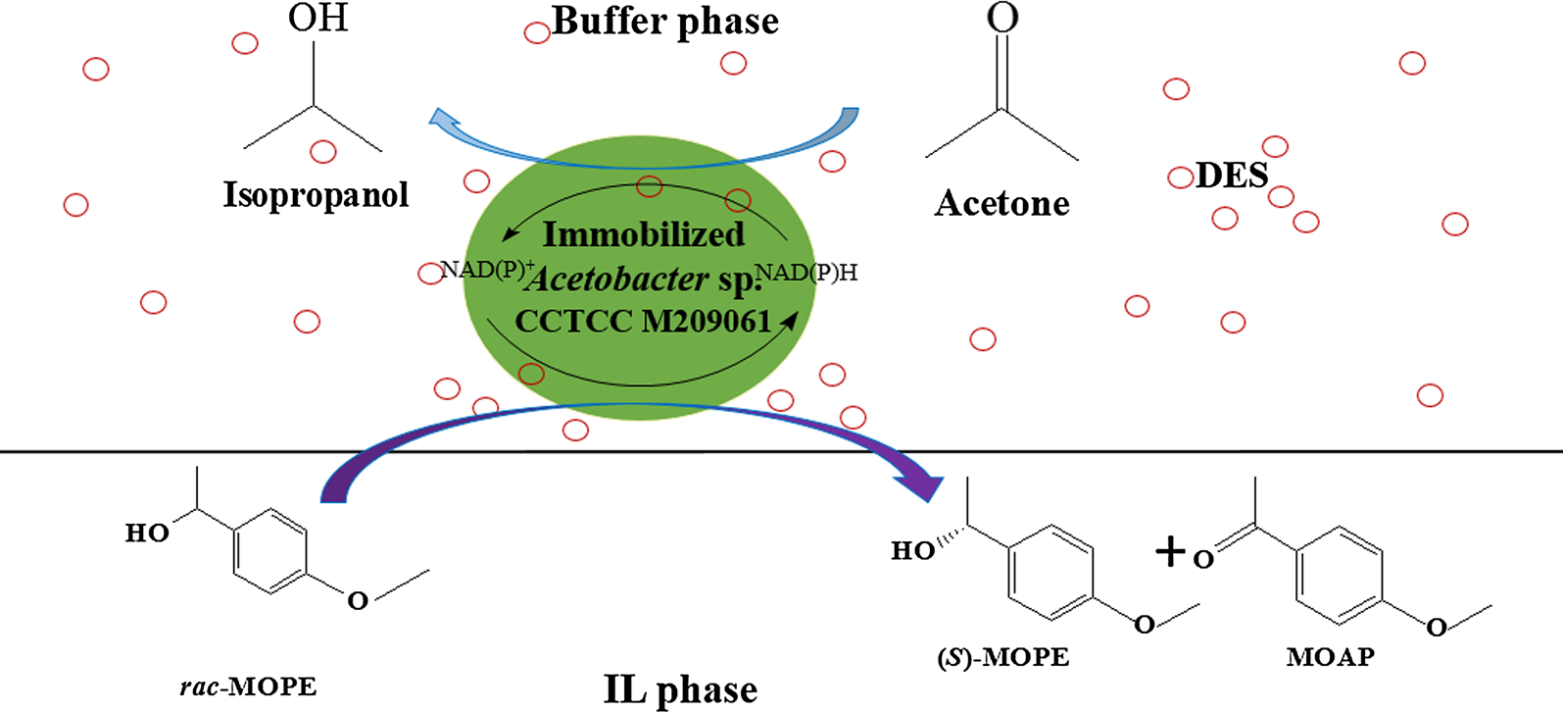
The asymmetric oxidation of racemic MOPE with immobilized *Acetobacter* sp. CCTCC M209061 cells in DES-containing biphasic system

In the present study, we have for the first time utilized various water-immiscible organic solvents and especially ionic liquids (ILs) as the second phase of a two-phase system to improve the biocatalytic resolution of racemic MOPE to obtain enantiopure (*S*)-MOPE through asymmetric oxidation, catalyzed by immobilized *Acetobacter* sp. CCTCC M209061 cells (Scheme 1). The biocompatibility of these ILs with *Acetobacter* sp. CCTCC M209061 and their effects on the biocatalytic reaction were explored systematically. Furthermore, adding DES to a water-immiscible IL-based biphasic system was examined for further enhancing the reaction efficiency of the biocatalytic oxidation of MOPE, and the efficient biocatalytic process was evaluated on a preparative scale.

## Results and discussion

### Effect of different water-immiscible organic solvents and ILs on the asymmetric oxidation of MOPE with immobilized *Acetobacter* sp. CCTCC M209061 cells

In many cases [27–29], the inhibition of substrate and/or product was unavoidable to the biocatalytic reaction in the aqueous system. Therefore, a biphasic system consisted of organic solvent or hydrophobic IL and buffer was conducted to improve the efficiency of the biocatalytic process. Many investigations have shown that the effects of different hydrophobic organic solvents and ILs on a biocatalytic reaction varied widely, and in many cases [26, 30, 31], the conversion/yield and the residual substrate or product *e.e.* would be enhanced significantly in the presence of the organic solvents or ILs compared to those in a aqueous monophasic phase. In this study, seven organic solvents and five hydrophobic ILs were selected to investigate the influence on the asymmetric oxidation of MOPE catalyzed by immobilized *Acetobacter* sp. CCTCC M209061 cells. As shown in Table 1, the initial reaction rate and conversion were improved apparently with the increasing hydrophobic of organic solvents (Log *P*). The immobilized cells emerged a better catalytic activity in *n*-hexane/buffer system among the surveyed seven organic solvents, with a higher initial reaction rate (51.3 μmol/min) and conversion yield (48.0 %). The more drastic hydrophobic of organic solvents, the higher initial rate and conversion were gained until the Log *P* reached 3.5 (*n*-hexane). When the Log *P* further increased, the initial reaction rate, the maximum conversion as well as the enantioselectivity decreased sharply, which might resulted from the stronger extraction of the phosphatide of the cytomembrane as the more hydrophobicity of the organic solvents. The fierce extraction led to inactivation of the microbial cells [32].

**Table 1:** Effect of various hydrophobic solvents on the asymmetric oxidation of MOPE catalyzed by *Acetobacter* sp. CCTCC M209061 cells

Hydrophobic solvents	Log *P*	Initial rate (μmol/min)	Reaction time (h)	Conversion^a^ (%)	*e.e.* ^b^ (%)
Aqueous buffer	–	75.0	10.0	47.5	91.7
Ethyl acetate/buffer	0.7	7.2	26.0	24.1	31.7
Isopropyl ether/buffer	1.9	16.8	24.0	36.0	56.3
Cyclohexane/buffer	3.0	45.0	13.0	47.2	89.0
*n*-hexane/buffer	3.5	51.3	12.0	48.0	92.3
Isooctane/buffer	3.9	42.5	15.0	45.5	83.4
*n*-nonane/buffer	4.7	34.9	18.0	42.7	74.5
Dodecane/buffer	6.6	30.3	20.0	41.0	69.5
[C_4_MIM][PF_6_]/buffer	–	59.3	12.0	48.5	95.1
[C_5_MIM] [PF_6_]/buffer	–	50.1	12.5	46.3	87.3
[C_2_MIM][Tf_2_N]/buffer	–	42.6	13.0	41.6	72.4
[C_4_MIM][Tf_2_N]/buffer	–	36.1	14.0	35.5	56.0
[PP_14_][Tf_2_N]/buffer	–	25.9	15.0	30.1	44.6

Reaction conditions: 3.5 mL TEA-HCl buffer (100 mmol/L, pH 6.5); 0.3 g/mL immobilized cells; 122 mmol/L acetone; 1.5 mL organic solvent containing 5.04 mmol/L *n*-tetradecane (as internal standard) or 1.5 mL hydrophobic IL; 50 mM MOPE; 30 °C; 200 rpm

^a^The maximum conversion

^b^The residue substrate *e.e.*

When the biocatalytic reaction is investigated in the IL/buffer system, the catalytic performance of the whole-cell is closely related to the cation and the anion types of ILs in various biphasic systems containing IL, and also the varied effects of ILs on the biocatalytic reactions have been found variously [33–35]. Therefore, five ILs have been investigated, in order to understand the influences of cations and anions in different ILs on such a reaction. Clearly shown in Table 1, When the cation was [C_n_MIM]^+^, and the anion was [PF_6_]^−^ or [Tf_2_N]^−^ in the biphasic reaction system, the initial reaction rate and the maximum conversion and the residual substrate *e.e.* decreased sharply with the length of the alkyl chain of the ILs’ cation (i.e. increasing *n* value), which could be ascribed to the increased viscosity and toxicity of these ILs with the increased *n* value. It was noted that, when the cation was [C_4_MIM]^+^ and [Tf_2_N]-based IL gave much lower initial reaction rate, conversion and residual substrate *e.e.* than the IL with [PF_6_]^−^. Moreover, the two [Tf_2_N]-based ILs biphasic systems, the bio-oxidation reaction efficiency changed as IL cation changed. The efficiency of the biocatlytic reaction was decelerated when the [PP_14_]^+^ replaced the [C_4_MIM]^+^ and the residual substrate *e.e.* was worst affected.

The best results were seen in [C_4_MIM][PF_6_]/buffer system of the five investigated IL/buffer systems and seven organic solvents, where the initial reaction rate and the maximum conversion of the asymmetric oxidation of racemic MOPE reached 59.3 μmol/min and 48.5 %, respectively, with the residual substrate *e.e.* of 95.1 %.

### Biocompatibility of organic solvents and ILs with *Acetobacter* sp. CCTCC M209061 cells

**Fig. 1:**
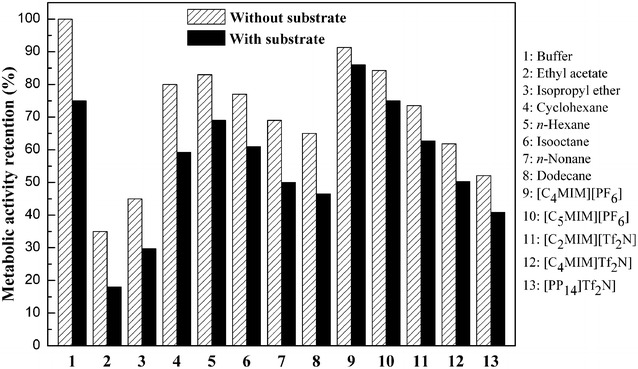
Effect of various organic solvents and ILs on glucose metabolic activity retention of *Acetobacter* sp. CCTCC M209061 cells. *Reaction conditions*: 2 mL TEA-HCl buffer (100 mmol/L, pH 6.5); 0.3 g/mL immobilized beads (cell wet weight 0.048 g/mL); 3 mL organic solvent or IL; 50 mmol/L MOPE; 30 °C; 200 rpm for 24 h; washed 3 times with deionized water; 10 g/L glucose incubated for 4 h; 30 °C

According to the existing studies [36, 37], the second phase has been found to be toxic to the biocatalysts, regardless of no matter organic solvents or ILs. Therefore, it is necessary to evaluate the biocompatibility of the used organic solvents and ILs by directly measuring the sugar metabolic activity retention (MAR, %) of *Acetobacter* sp. CCTCCM209061 cell, which generally depends on its tolerance to solvents and is taken as an easy indicator of cell viability [37, 38], after 24 h exposure to the two-phase systems involving various ILs and organic solvents, in the absence and presence of substrate. As shown in Fig. 1, the MAR value of the *Acetobacter* sp. CCTCCM209061 cells was lower in all the tested organic solvents and ILs biphasic systems than that in aqueous system in the absence of MOPE, suggesting that the examined organic solvents and ILs were all toxic to *Acetobacter* sp. CCTCCM209061 cells to some extent. The MAR value varied obviously in the investigated organic solvents and hydrophobic ILs, of which [C_4_MIM][PF_6_] exhibited the best biocompatibility with the microbial cells and giving the highest MAR value of 92 %. Also, it was noteworthy that in the existence of MOPE (50 mmol/L) the MAR value of the microbial cells after incubation decreased clearly in all the tested systems as compared with that without MOPE, which possibly resulting from the toxicity of the substrate MOPE to *Acetobacter* sp. CCTCCM209061 cells. Of all reaction media, the highest MAR value of the microbial cells (86 %) at the present of MOPE was observed in [C_4_MIM][PF_6_]/buffer system. This agreed with the fastest initial reaction rate, the maximum conversion achieved in the [C_4_MIM][PF_6_]-based biphasic system. Interestingly noticed that, the MAR value with MOPE was reduced only 6 % relative to that without MOPE in the [C_4_MIM][PF_6_]/buffer system, which was much less than that (abound 25 %) in aqueous system, indicating that the [C_4_MIM][PF_6_]-based biphasic system could not only have the good biocompatibility to the *Acetobacter* sp. CCTCCM209061 cells, and also have excellent extraction to MOPE. As a result, the [C_4_MIM][PF_6_] was selected as the second phase in the biphasic system for the asymmetric oxidation of racemic MOPE.

### Effects of several key variables on the asymmetric oxidation of racemic MOPE with immobilized *Acetobacter* sp. CCTCCM209061 in biphasic system

The above-described results clearly showed that the [C_4_MIM][PF_6_]/buffer biphasic system was the potential reaction medium for the asymmetric oxidation of racemic MOPE catalyzed by immobilized *Acetobacter* sp. CCTCC M209061 cells. And consequently the biocatalytic process was systematically optimized in the existence of [C_4_MIM][PF_6_] to further improve the biotransformation with respect to the initial reaction rate, the maximum conversion and the residual substrate *e.e.* on the basis of several crucial variables such as [C_4_MIM][PF_6_] content, reaction temperature, buffer pH, substrate concentrations.

It has been demonstrated that the amount of IL in a biphasic system affects significantly on the activity, enantioselectivity and stability of enzymes and microbial cells [39, 40]. Therefore, it is necessary to investigate the effect of [C_4_MIM][PF_6_] content in the biphasic system. As depicted in Table 2, the content of the [C_4_MIM][PF_6_] in the IL/buffer biphasic system displayed significant influence on the biocatalytic reaction. The initial reaction rate increased drastically as the occupancy volume of [C_4_MIM][PF_6_] increased from 12 to 20 %, and the maximum conversion as well as the residual substrate *e.e.* also went up to some extent. However, further increased the content of [C_4_MIM][PF_6_] resulted in a clear drop in the initial reaction rate, the conversion and the residual substrate *e.e*. The increase in the initial reaction rate and the maximum conversion with the raised [C_4_MIM][PF_6_] content up to 20 % may be accounted for the improved membrane permeability, which led to the enhance of mass transfer of the substrate and the product to and from the immobilized cells at a proper [C_4_MIM][PF_6_] content. However, the decline in the initial rate and the conversion at higher [C_4_MIM][PF_6_] content could due to the increased viscosity of the system, limiting the mass transfer, and the higher level of IL toxicity to the cells. Therefore, the optimal [C_4_MIM][PF_6_] content was considered as 20 %.

**Table 2:** Effect of [C_4_MIM][PF_6_] concentration on the asymmetric oxidation of MOPE catalyzed by *Acetobacter* sp. CCTCC M209061 cells

[C_4_MIM][PF_6_] concentration (%)	Initial rate (μmol/min)	Reaction time (h)	Conversion^a^ (%)	*e.e.* ^b^ (%)
12	57.3	12.5	48.0	93.4
18	67.7	12.0	49.1	97.5
20	75.0	11.0	50.7	99.9
22	68.4	11.5	49.6	99.4
25	59.3	12.0	48.5	95.1
30	50.9	13.0	47.2	90.4

Reaction conditions: 5 mL different ratio [C_4_MIM][PF_6_] and TEA-HCl buffer (100 mmol/L, pH 6.5); 0.3 g/mL immobilized cells; 80 mmol/L acetone; 50 mmol/L MOPE; 30 °C; 200 rpm

^a^The maximum conversion

^b^The residue substrate *e.e.*

**Fig. 2:**
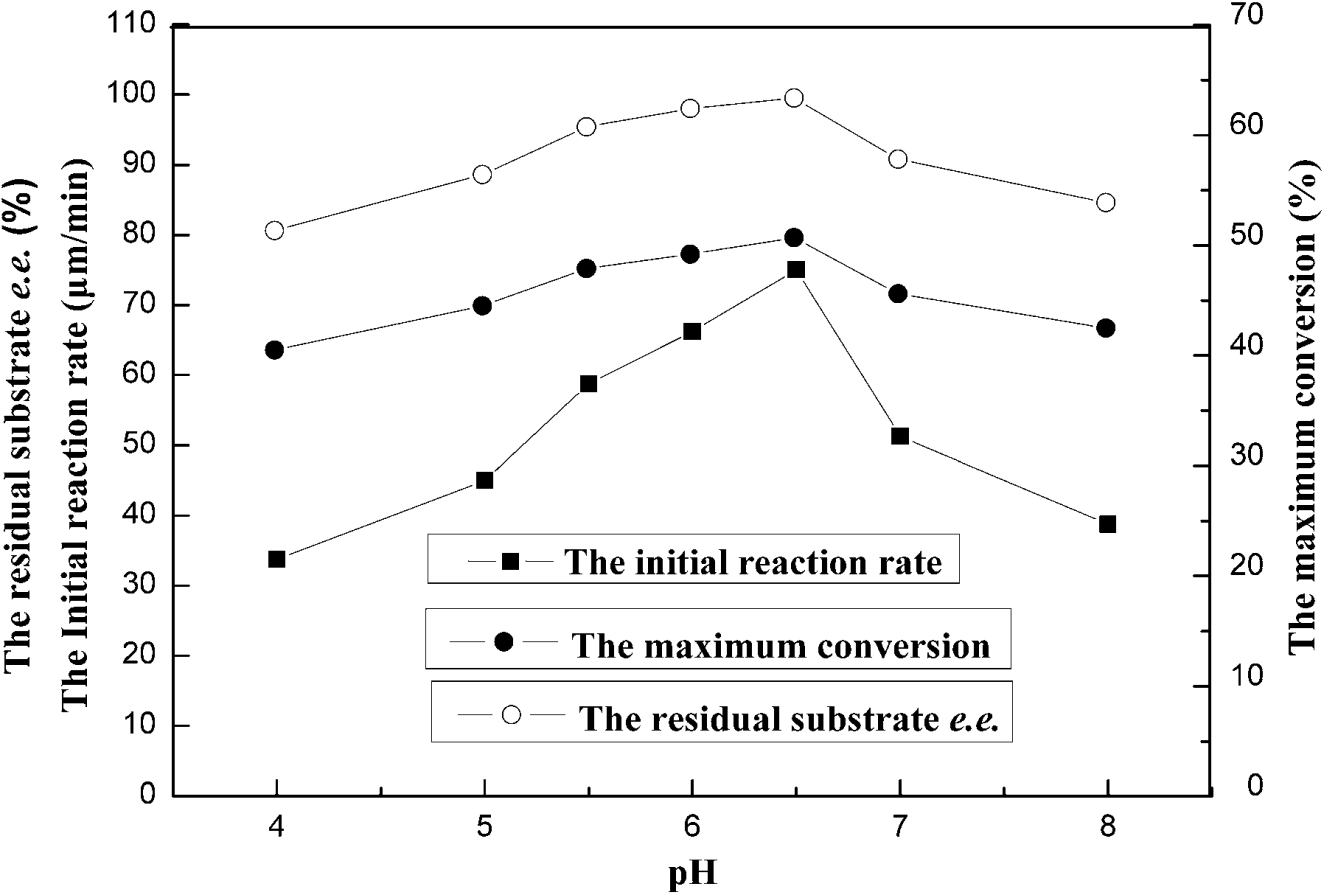
Effect of buffer pH on the asymmetric oxidation of MOPE in [C_4_MIM][PF_6_]/buffer biphasic system. Reaction conditions: 4 mL TEA-HCl buffer (100 mmol/L, pH 4.0–8.0); 1 mL [C_4_MIM][PF_6_]; 50 mmol/L MOPE; 0.3 g/mL immobilized beads; 80 mmol/L acetone; 30 °C; 200 rpm

Diverse pH values could not only influence the activity and the selectivity of the biocatalyst, but also the recycle of the coenzyme existing in the microbial cells, which in turn affects the reaction rate distinctly [41]. Hence, the effects of various pHs (4.0-8.0) on the activities of immobilized *Acetobacter* sp. CCTCC M209061cells were examined for the asymmetric oxidation of racemic MOPE. As illustrated in Fig. 2, raising buffer pH from 4.0 to 6.5 gave a rise to an increase in the initial rate and the maximum conversion, and there was almost no change in the residual substrate *e.e.* (>99 %). While further rising in buffer pH from 6.5 to 8.0 led to a clear drop in the initial rate and the maximum conversion. Thus, the optimal buffer pH was shown to be 6.5.

**Fig. 3:**
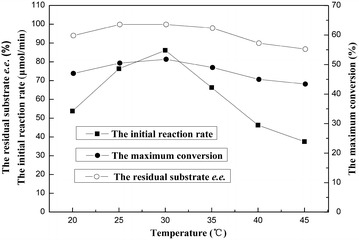
Effect of temperature on the asymmetric oxidation of MOPE in [C_4_MIM][PF_6_]/buffer biphasic system. Reaction conditions: [C_4_MIM][PF_6_]/buffer biphasic system: 4 mL TEA-HCl buffer (100 mmol/L, pH 6.5), 1 mL [C_4_MIM][PF_6_]; 50 mmol/L MOPE; 0.3 g/mL immobilized beads; 80 mmol/L acetone; 20–45 °C; 200 rpm

Reaction temperature can significantly affect the selectivity and stability of the whole-cell catalyst, as well as the equilibrium of a reaction [42]. Therefore, it is necessary to examine the influences of different reaction temperature on the asymmetric oxidation process. As shown in Fig. 3, the oxidation reaction proceeded faster and the maximum conversion increased with raising temperature up from 20 to 30 °C. Further rise in temperature led to a clear drop in the initial reaction rate, the maximum conversion as well as the residual substrate *e.e.*, which could be attributed to the partial inactivation of the microbial cells. Thence, the optimal temperature for the reaction was considered to be 30 °C.

Table 3 described the dramatic influence of substrate concentration on the reaction in [C_4_MIM][PF_6_]/buffer system. The initial reaction rate increased markedly from 89.2 to 97.8 μmol/min with the change of MOPE concentration from 55 to 65 mmol/L, while the maximum conversion showed a little decrease, and there was almost no change in the residual substrate *e.e.* (>99.9 %). Further increasing the substrate concentration from 65 to 80 mmol/L resulted in a significant drop in the initial reaction rate, possibly due to the growing substrate inhibition on the immobilized *Acetobacter* sp. CCTCC M209061 cells. Consequently, the optimal substrate concentration in the [C_4_MIM][PF_6_]/buffer system was regarded as 65 mmol/L.

**Table 3:** Effect of substrate concentration on the asymmetric oxidation of MOPE in [C_4_MIM][PF_6_]/buffer biphasic system

Substrate concentration (mmol/L)	Initial reaction rate (μmol/min)	Reaction time (h)	Conversion^a^ (%)	*e.e.* ^b^ (%)
55.0	89.2	11.0	51.8	99.9
60.0	93.6	10.5	51.1	99.9
65.0	97.8	10.0	50.5	99.9
70.0	95.9	11.0	49.4	98.6
75.0	84.5	12.0	48.8	96.4
80.0	74.1	13.0	47.5	91.5

Reaction conditions: 4 mL TEA-HCl buffer (100 mmol/L, pH 6.5); 0.3 g/mL immobilized cells; 122 mmol/L acetone; 1 mL [C_4_MIM][PF_6_]; 55–80 mmol/L MOPE; 30 °C; 220 rpm

^a^The maximum conversion

^b^The residue substrate *e.e.*

### Effect of adding [ChCl][Gly] for enhancing the substrate concentration of MOPE in the biphasic system

**Fig. 4:**
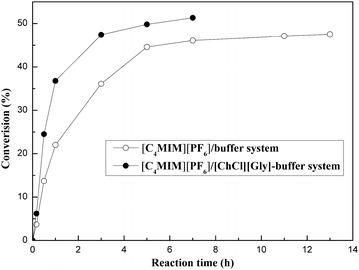
Biocatalytic process of the asymmetric oxidation of MOPE with Acetobacter sp. CCTCC M209061 strain in different reaction systems. *Reaction conditions:* (1) [C_4_MIM][PF_6_]/buffer biphasic system: 4 mL TEA-HCl buffer (100 mmol/L, pH 6.5); 1 mL [C_4_MIM][PF_6_]; 80 mmol/L MOPE; 0.3 g/mL immobilized beads; 122 mmol/L acetone; 30 °C; 220 rpm. (2) [C_4_MIM][PF_6_]/[ChCl][Gly]-buffer biphasic system:3.6 mL TEA-HCl buffer (100 mmol/L, pH 6.5); 1 mL [C_4_MIM][PF_6_]; 0.4 mL [ChCl][Gly]; 80 mmol/L MOPE, 0.3 g/mL immobilized beads; 122 mmol/L acetone; 30 °C; 220 rpm

Though hydrophobic ILs/buffer biphasic system could effectively alleviate the substrate or product inhibition, the initial reaction rate was lower than that in aqueous system that will prolong the reaction time [43, 44], which motivated us to find a new reagent to further improve the efficiency of asymmetric oxidation reaction. Existing studies showed that the addition of the water-miscible DES into an aqueous system was able to expedite the biocatalytic reaction [26, 45]. Therefore, it was of great interest to combine water-miscible DES with water-immiscible IL to seriously improve the efficiency of asymmetric oxidation of racemic MOPE with the immobilized *Acetobacter* sp. CCTCC M209061cells. Based on our previous study [26], a kind of DES ([ChCl][Gly]) was appended in the biphasic system, which occupancy volume was 10 % of the buffer to ameliorate the catalytic oxidation process. When enhanced the concentration of MOPE regularly, the initial rate was increased constantly, until the substrate concentration reached 80 mmol/L in the existence of [ChCl][Gly] [C_4_MIM][PF_6_]/buffer biphasic system. As shown in Table 4, the maximum conversion and the residual substrate *e.e.* were 51.3 and >99.9 %, respectively. Compared with the [C_4_MIM][PF_6_]/buffer system, the introduction of [ChCl][Gly] to the reaction system was improved the substrate concentration from 65 to 80 mmol/L, shortening the reaction from 10 h to 7 h with a higher initial rate (97.8 μmol/min VS 124.0 μmol/min). The influence of [ChCl][Gly] might result mostly by the improved cell membrane permeability, which expedited the mass transfer, thus giving rise to a higher initial reaction rate, reducing the toxic and inhibitory effects of the substrate as well as the product and limiting the reverse reaction [45]. As shown in the Fig. 4, when the concentration of racemic MOPE was 80 mmol/L, the asymmetric oxidation with the immobilized cells were evaluated in [C_4_MIM][PF_6_]/buffer system and [C_4_MIM][PF_6_]/[ChCl][Gly]-containing buffer system, respectively. Compared with the reaction without [ChCl][Gly] in the biphasic system, the initial rate (74.1 μmol/min vs 124.0 μmol/min) and the maximum conversion (47.5 % vs 51.3 %) was increased rapidly, and the reaction process was curtate for 6 h in the [C_4_MIM][PF_6_]/[ChCl][Gly]-containing buffer system, which encouraged us to the further study about the operational stability of the immobilized *Acetobacter* sp. CCTCC M209061 cells and preparative scale biocatalytic asymmetric oxidation of racemic MOPE by the immobilized *Acetobacter* sp. CCTCC M209061 cells in the [C_4_MIM][PF_6_]/[ChCl][Gly]-containing buffer system.

**Table 4:** Effect of substrate concentration on the asymmetric oxidation of MOPE in C_4_MIM·PF_6_/[ChCl][Gly]- buffer biphasic system

Substrate concentration (mmol/L)	Initial reaction rate (μmol/min)	Reaction time (h)	Conversion^a^ (%)	*e.e.* ^b^ (%)
70.0	90.6	9.0	51.8	99.9
75.0	94.9	8.5	51.1	99.9
80.0	124.0	7.0	51.3	99.9
85.0	107.2	10.0	49.4	98.6
90.0	95.8	11.0	48.8	96.4
95.0	85.4	12.0	47.5	91.5

Reaction conditions: 4 mL TEA-HCl buffer (100 mmol/L, pH 6.5); 0.3 g/mL immobilized cells; 122 mmol/L acetone; 1 mL [C_4_MIM][PF_6_]; 70–95 mmol/L MOPE; 30 °C; 220 rpm

^a^The maximum conversion

^b^The residue substrate *e.e.*

### Operational stability of immobilized *Acetobacter* sp. CCTCC M209061 cells

**Fig. 5:**
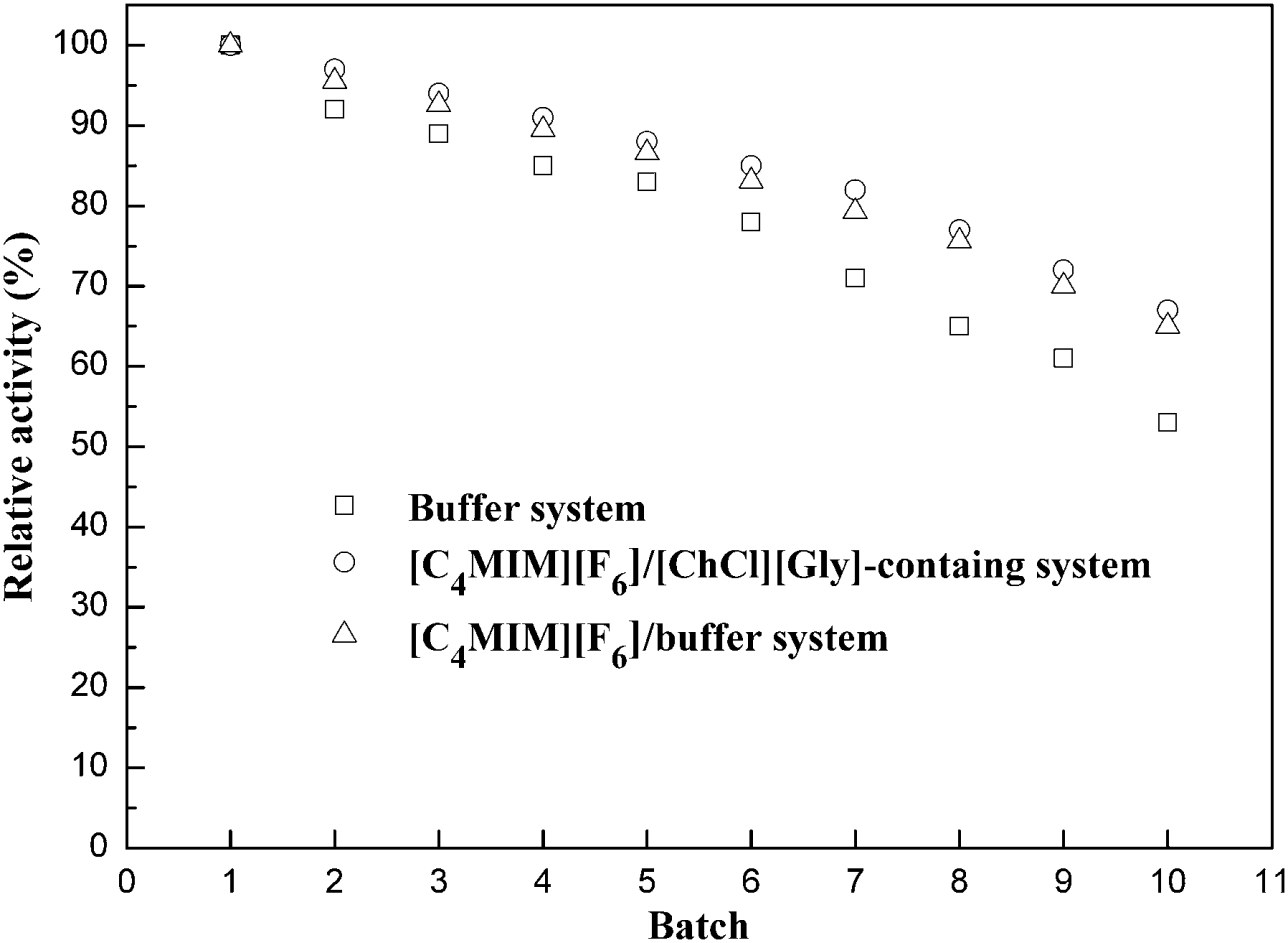
Operational stability of immobilized *Acetobaceter* sp. CCTCC M209061 cells in various reaction systems. Reaction conditions: (1) aqueous buffer system: 500 mL TEA-HCl buffer (100 mmol/L, pH 6.5); 30 mmol/L MOPE; 0.3 g/mL immobilized beads; 70 mmol/L acetone; 30 °C; 180 rpm. (2) [C_4_MIM][PF_6_]/buffer biphasic system: 400 mL TEA-HCl buffer (100 mmol/L, pH 6.5), 100 mL [C_4_MIM][PF_6_]; 65 mmol/L MOPE; 0.3 g/mL immobilized beads; 122 mmol/L acetone; 30 °C; 220 rpm. (3) [C_4_MIM][PF_6_]/[ChCl][Gly]-containing buffer biphasic system:360 mL TEA-HCl buffer (100 mmol/L, pH 6.5); 100 mL [C_4_MIM][PF_6_]; 40 mL [ChCl][Gly]; 80 mmol/L MOPE; 0.3 g/mL immobilized beads; 122 mmol/L acetone; 30 °C; 220 rpm

To evaluate the operational stability of the immobilized *Acetobacter* sp. CCTCC M209061 cells, the batch reuse of the immobilized cells was investigated in the various reaction systems under the optimized reaction conditions. Between each cycle of the reaction, the immobilized cells were recovered by filtration, washed with water, and then reused in the next run. As shown in Fig. 5, the immobilized cells showed superior retention of activity in [C_4_MIM][PF_6_]/[ChCl][Gly]-containing buffer system compared to that in aqueous system and [C_4_MIM][PF_6_]/buffer system, the relative activity remained around 72.0 % after 9 batches, which revealed a favourable application prospect.

### Preparative scale biocatalytic oxidation of MOPE in various reaction systems

To test the applicability of the biocatalytic asymmetric oxidation of MOPE to obtain (*S*)-MOPE using immobilized *Acetobacter* sp.CCTCC M209061 cells in various reaction systems, the bio-oxidation on 500 mL preparative scale was carried out. The reaction process was monitored by GC analysis, and the reactants were extracted from the reaction mixture with isopropyl ether when no (*R*)-MOPE was detected. The initial reaction rate (111.3 μmol/min) and the maximum conversion (50.2 %) of the asymmetric oxidation in the [C_4_MIM][PF_6_]/[ChCl][Gly]-containing buffer biphasic system was slightly lower than that in 5-mL scale after reaction for 7 h, but the residual substrate *e.e.* still above 99.9 %. Obviously, the reaction efficiency of the asymmetric oxidation of racemic MOPE in the [C_4_MIM][PF_6_]/[ChCl][Gly]-containing buffer biphasic system was much higher than that in aqueous system and [C_4_MIM][PF_6_]/buffer system (shown in Table 5). Hence, the immobilized *Acetobacter* sp. CCTCC M209061 cell-catalyzed asymmetric oxidation of racemic MOPE on a preparative scale [C_4_MIM][PF_6_]/[ChCl][Gly]-containing buffer biphasic system was promising and competitive.

**Table 5:** Preparative scale oxidation of racemic MOPE catalyzed by *Acetobacter* sp. CCTCC M209061 cells in various reaction systems

Reaction media	Optimal substrate concentration (mmol/L)	Initial rate (μmol/min)	Reaction time (h)	Conversion^a^ (%)	*e.e.* ^b^ (%)
Buffer	30.0	95.0	9.0	45.6	85.0
C_4_MIM·PF_6_/buffer	65.0	90.3	10.0	49.2	97.9
C_4_MIM·PF_6_/[ChCl][Gly]-containing buffer	80.0	113.3	7.0	50.2	99.9

Reaction conditions: (1) aqueous buffer system: 500 mL TEA-HCl buffer (100 mmol/L, pH 6.5); 30 mmol/L MOPE, 0.3 g/mL immobilized beads; 70 mmol/L acetone; 30 °C; 180 rpm. (2) [C_4_MIM][PF_6_]/buffer system: 400 mL TEA-HCl buffer (100 mmol/L, pH 6.5), 100 mL [C_4_MIM][PF_6_]; 65 mmol/L MOPE; 0.3 g/mL immobilized beads; 122 mmol/L acetone; 30 °C; 220 rpm. (3) [C_4_MIM][PF_6_]/[ChCl][Gly]-containing buffer system: 360 mL TEA-HCl buffer (100 mmol/L, pH 6.5); 100 mL [C_4_MIM][PF_6_]; 40 mL [ChCl][Gly]; 80 mmol/L MOPE; 0.3 g/mL immobilized beads; 122 mmol/L acetone; 30 °C; 220 rpm

^a^The maximum conversion

^b^The residual substrate *e.e.*

## Conclusion

The biocatalytic resolution of racemic MOPE to get enantiopure (S)-MOPE was successfully performed with high conversion and enantioselectivity through asymmetric oxidation of MOPE catalyzed by *Acetobacter* sp. CCTCC M209061 cells in a two-phase system. The examined water-immiscible ILs and organic solvents as the second phase of a two-phase system manifested significant but different effects on the microbial cell-based oxidation reaction. Of all these solvents, the IL [C_4_MIM][PF_6_] showed better biocompatibility with the microbial cells and presented the best biotransformation results. Furthermore, the reaction efficiency of the biocatalytic oxidation of MOPE was further enhanced by adding the DES [ChCl][Gly] to the [C_4_MIM][PF_6_]-based biphasic system. The immobilized cells indicated excellent operational stability in the reaction system. Also, the biocatalytic process was feasible up to a 500-mL preparative scale. Obviously, the efficient biocatalytic asymmetric oxidation of MOPE in the biphasic system involving [C_4_MIM][PF_6_] and [ChCl][Gly] was promising.

## Methods

### Biological and chemical materials

The strain of *Acetobacter* sp. CCTCC M209061 used in the experiment was isolated from Chinese kefir grains by our research group and conserved in our laboratory [10].

Racemic MOPE (98 % purity) was purchased from Alfa Aesar (USA). 4′-Methoxyacetophenone (99 %) and *n*-tetradecane (>99 %) were purchased from TCI (Japan). The five ILs used in this work, 1-butyl-3-methylimidazolium hexafluorophoshpate ([C_4_MIM][PF_6_]), 1-pentyl-3-methylimidazolium hexafluorophoshpate ([C_5_MIM][PF_6_]), 1-ethyl-3-methylimidazolium bis(trifluoromethanesulfonyl)imide ([C_2_MIM][Tf_2_N]), 1-butyl-3-methylimidazolium bis(trifluoromethanesulfonyl)imide ([C_4_MIM][Tf_2_N]), *N*-butyl-*N*-methylpiperidinium bis(trifluoromethanesulfonyl)imide ([PP_14_][Tf_2_N]) were purchased from Lanzhou Institute of Chemical Physics (China) with a purity of >98 %. All other reagents and solvents were of analytical grade and used without further purification.

### Cultivation and immobilization of *Acetobacter* sp. CCTCC M209061 cells

*Acetobacter* sp. CCTCC M209061 was cultivated according to our previous described methods [46]. The wet cells of *Acetobacter* sp. CCTCC M209061 were immobilized before used in the asymmetric oxidation, and the immobilization via chitosan entrapment and cross-linked by glyoxal and tetrasodium pyrophosphate mixing solution [26]. In a short, a homogenous cell/chitosan suspension was prepared at 25 °C by adding 6 g of fresh cells suspension (3 g wet cells in 3 mL water) into 47 mL of a homogeneous aqueous chitosan solution [3 %, w/v; prepared by dissolving chitosan in acetate buffer (pH 4.2)], heating and ultrasonic processing (20 kHz, 30 min). The suspension was added dropwise by a syringe into the cross-linking solution, which was mixed by 4 % (w/v) glyoxal solution and an equal volume of 3 % (w/v) tetrasodium pyrophosphate solution (pH 8.0). The immobilized beads were placed at room temperature for 30 min to harden. Then the beads were transferred to 0.05 % glutaraldehyde for reinforcement treatment [47, 48]. Collect the immobilized cells and washed the immobilized cells with sterilized water to remove the residual solution. The capacity of *Acetobacter* sp. CCTCC M209061 cells (based on cell wet mass) on the beads was 15 % (w/w). The resulting beads were stored in triethanolamine (TEA)-HCl buffer (100 mmol/L, pH 6.5) at 4 °C for later use.

### General procedure for biocatalytic asymmetric oxidation of MOPE

In a typical experiment, a system (5.0 mL) consisted of 1.5 mL organic solvent (containing 5.04 mmol/L *n*-tetradecane as an internal standard) or hydrophobic IL and 3.5 mL TEA–HCl buffer (100 mmol/L, pH 6.5) added to a 10 mL Erlenmeyer flask capped with a septum. And then 0.3 g/mL immobilized cells as well as 80 mmol/L acetone (the concentration was based on the reaction system) were added to the system. The reaction mixture was pre-incubated at an appropriate temperature and shaking rare for 15 min and then was initiated by adding MOPE (precalculated concentration, based on the volume of the entire solvent system) to the reaction system. Samples (50 μL) from organic phase or IL phase were taken regularly. The samples from organic phase can be tested for GC analysis directly, and product and residual substrate should be extracted from the samples of IL phase with 100 μL isopropyl (2 × 50 μL) containing 5.04 mmol/L *n*-tetradecane (internal standard). The details of the reaction were specified for each case.

### Metabolic activity retention measurement

The metabolic activity retention (MAR, %) of immobilized *Acetobacter* sp. CCTCC M209061 cells was defined as the ratio of the consumed glucose amount by the immobilized cells pretreated in various media to that by the immobilized cells pretreated in aqueous buffer (as the control) [49, 50]. The MAR of immobilized *Acetobacter* sp. CCTCC M209061 cells was tested after 24 h exposure in various systems consisting of 3 mL different organic solvents or hydrophobic ILs and 2 mL TEA-HCl buffer (100 mmol/L, pH 6.5), or in 5 mL TEA-HCl buffer (100 mmol/L, pH 6.5) system in the presence or in the absence of substrate (50 mmol/L MOPE, based on the volume of the entire solvent system), respectively. The immobilized *Acetobacter* sp. CCTCC M209061 cells were added to each system with the final concentration being 0.3 g/mL, and then incubated (30 °C, 200 rpm) for 24 h. After the incubation, the immobilized cells were collected by filtration, washed three times with distilled water, transferred to glucose solution (10 mL, 10.0 g/L), and then incubated at 30 °C and 200 rpm for 4 h. The glucose concentration in the medium was then assayed by HPLC.

### Analytical methods

The product and the residual substrate were analyzed by GC (Shimadzu GC 2010) as reported previously [26]. The retention times for *n*-tetradecane, MOAP, (*R*)-MOPE and (*S*)-MOPE were 3.3, 4.4, 5.4 and 5.6 min, respectively. The glucose concentration was assayed by high-performance liquid chromatography (HPLC) as described previously [14]. The average error for these determinations were <1.0 %. All reported data are averages of experiments performed at least in duplicate.

## Abbreviations

MOPE: 1-(4-methoxyphenyl) ethanol
MOAP: 4′-methoxyacetophenone
(*S*)-MOPE: (*S*)-1-(4-methoxyphenyl) ethanol
(*R*)-MOPE: (*R*)-1-(4-methoxyphenyl) ethanol
MAR: Metabolic activity retention
DESs: Deep eutectic solvents
ILs: Ionic liquids
TEA-HCl buffer: A kind of buffer prepared by triethanolamine and HCl
[C_4_MIM][PF_6_]: 1-Butyl-3-methylimidazolium hexafluorophoshpate
[C_5_MIM][PF_6_]: 1-Pentyl-3-methylimidazolium hexafluorophoshpate
[C_2_MIM][Tf_2_N]: 1-Ethyl-3-methylimidazolium bis(trifluoromethanesulfonyl)imide
[C_4_MIM][Tf_2_N]: 1-Butyl-3-methylimidazolium bis(trifluoromethanesulfonyl)imide
[PP_14_][Tf_2_N]: *N*-butyl-*N*-methylpiperidinium bis(trifluoromethanesulfonyl)imide
[ChCl][Gly]: A kind of deep eutectic solvent prepared by choline chloride and glycerol

## References

[1] Zilbeyaz K, Kurbanoglu EB (2008). Production of (*R*)-1-(4-bromo-phenyl)-ethanol by locally isolated* Aspergillus niger* using ram horn peptone. Bioresour Technol.

[2] Goldberg K, Schroer K, Lutz S, Liese A (2007). Biocatalytic ketone reduction—a powerful tool for the production of chiral alcohols—part II: whole-cell reductions. Appl Microbiol Biot.

[3] Hillier MC, Marcoux JF, Zhao DL, Grabowski EJJ, McKeown AE, Tillyer RD (2005). Stereoselective formation of carbon-carbon bonds via S(N)2-displacement: synthesis of substituted cycloalkyl b indoles. J Org Chem.

[4] Hillier MC, Desrosiers JN, Marcoux JF, Grabowski EJJ (2004). Stereoselective carbon-carbon bond formation via the mitsunobu displacement of chiral secondary benzylic alcohols. Org Lett.

[5] Lou WY, Wang W, Smith TJ, Zong M-H (2009). Biocatalytic anti-Prelog stereoselective reduction of 4′-methoxyacetophenone to (*R*)-1-(4-methoxyphenyl)ethanol with immobilized Trigonopsis variabilis AS2.1611 cells using an ionic liquid-containing medium. Green Chem.

[6] Zong MH, Wang W, Lou WY (2008). Efficient asymmetric reduction of 4′-methoxyacetophenone catalyzed by immobilized Trigonopsis variabilis cells using ionic liquid-containing systems. J Biotechnol.

[7] Wang W, Zong MH, Lou WY. Use of an ionic liquid to improve asymmetric reduction of 4′-methoxyacetophenone catalyzed by immobilized *Rhodotorula* sp. AS2.2241 cells. J Mol Catal B Enzym. 2009;56:70–6.

[8] Lou WY, Wang W, Li RF, Zong M-H (2009). Efficient enantioselective reduction of 4′-methoxyacetophenone with immobilized *Rhodotorula* sp. AS2.2241 cells in a hydrophilic ionic liquid-containing co-solvent system. J Biotechnol.

[9] Cheng J, Lou WY, Zong MH (2014). Biocatalytic asymmetric oxidation of racemic 1-(4-Methoxyphenyl) ethanol using immobilized *Acetobacter* sp. CCTCC M209061 cells in organic solvent-containing biphasic system. Chem J Chin U.

[10] Xiao ZJ, Zong MH, Lou WY (2009). Highly enantioselective reduction of 4-(trimethylsilyl)-3-butyn-2-one to enantiopure (*R*)-4-(trimethylsilyl)-3-butyn-2-ol using a novel strain *Acetobacter* sp. CCTCC M209061. Bioresour Technol.

[11] Hussain W, Pollard DJ, Lye GJ (2007). The bioreduction of a beta-tetralone to its corresponding alcohol by the yeast Trichosporon capitatum MY1890 and bacterium Rhodococcus erythropolis MA7213 in a range of ionic liquids. Biocatal Biotransfor.

[12] Braeutigam S, Dennewald D, Schuermann M, Lutje-Spelberg J, Pitner WR, Weuster-Botz D (2009). Whole-cell biocatalysis: evaluation of new hydrophobic ionic liquids for efficient asymmetric reduction of prochiral ketones. Enzyme Microbial Technol.

[13] Wang XT, Chen XH, Xu Y, Lou WY, Wu H, Zong MH (2013). Biocatalytic anti-Prelog stereoselective reduction of ethyl acetoacetate catalyzed by whole cells of *Acetobacter* sp. CCTCC M209061. J Biotechnol.

[14] Wang XT, Yue DM, Zong MH, Lou WY (2013). Use of ionic liquid to significantly improve asymmetric reduction of ethyl acetoacetate catalyzed by *Acetobacter* sp. CCTCC M209061 Cells. Ind Eng Chem Res.

[15] He JY, Sun ZH, Ruan WQ, Xu Y (2006). Biocatalytic synthesis of ethyl (*S*)-4-chloro-3-hydroxy-butanoate in an aqueous-organic solvent biphasic system using Aureobasidium pullulans CGMCC 1244. Process Biochem.

[16] Xu P, Zheng GW, Du PX, Zong MH, Lou WY (2015). Whole-cell biocatalytic processes with ionic liquids. ACS Sustain Chem Eng.

[17] Abo-Hamad A, Hayyan M, AlSaadi MA, Hashim MA (2015). Potential applications of deep eutectic solvents in nanotechnology. Chem Eng J.

[18] Zhao H (2015). DNA stability in ionic liquids and deep eutectic solvents. J Chem Technol Biot.

[19] Smith EL, Abbott AP, Ryder KS (2014). Deep eutectic solvents (DESs) and their applications. Chem Rev.

[20] Zhang Q, Vigier KDO, Royer S, Jerome F (2012). Deep eutectic solvents: syntheses, properties and applications. Chem Soc Rev.

[21] Shahbaz K, Mjalli FS, Hashim MA, AlNashef IM (2011). Using deep eutectic solvents based on methyl triphenyl phosphunium bromide for the removal of glycerol from palm-oil-based biodiesel. Energ Fuel.

[22] Gorke JT, Srienc F, Kazlauskas RJ. Hydrolase-catalyzed biotransformations in deep eutectic solvents. Chem Commun. 2008:1235–7. 10.1039/b716317g 18309428

[23] Huang ZL, Wu BP, Wen Q, Yang TX, Yang Z (2014). Deep eutectic solvents can be viable enzyme activators and stabilizers. J Chem Technol Biot.

[24] Durand E, Lecomte J, Barea B, Piombo G, Dubreucq E, Villeneuve P (2012). Evaluation of deep eutectic solvents as new media for Candida antarctica B lipase catalyzed reactions. Process Biochem.

[25] Mueller CR, Lavandera I, Gotor-Fernandez V, Dominguez de Maria P. Performance of recombinant-whole-cell-catalyzed reductions in deep-eutectic-solvent-aqueous-media mixtures. Chemcatchem. 2015;7:2654–9.

[26] Xu P, Cheng J, Lou WY, Zong MH (2015). Using deep eutectic solvents to improve the resolution of racemic 1-(4-methoxyphenyl)ethanol through *Acetobacter* sp. CCTCC M209061 cell-mediated asymmetric oxidation. Rsc Adv.

[27] Coronel C, Arce G, Iglesias C, Noguera CM, Bonnecarrere PR, Giordano SR, Gonzalez D. Chemoenzymatic synthesis of fluoxetine precursors. Reduction of beta-substituted propiophenones. J Mol Catal B Enzym. 2014;102:94–8.

[28] Yu CY, Wei P, Li XF, Zong MH, Lou WY (2014). Using ionic liquid in a biphasic system to improve asymmetric hydrolysis of styrene oxide catalyzed by cross-linked enzyme aggregates (CLEAs) of mung bean epoxide hydrolases. Ind Eng Chem Res.

[29] Lou WY, Chen L, Zhang BB, Smith TJ, Zong MH (2009). Using a water-immiscible ionic liquid to improve asymmetric reduction of 4-(trimethylsilyl)-3-butyn-2-one catalyzed by immobilized Candida parapsilosis CCTCC M203011 cells. BMC Biotechnol.

[30] Chen WJ, Lou WY, Zong MH (2012). Efficient asymmetric hydrolysis of styrene oxide catalyzed by Mung bean epoxide hydrolases in ionic liquid-based biphasic systems. Bioresour Technol.

[31] Yu CY, Li XF, Lou WY, Zong MH (2013). Cross-linked enzyme aggregates of Mung bean epoxide hydrolases: a highly active, stable and recyclable biocatalyst for asymmetric hydrolysis of epoxides. J Biotechnol.

[32] Nakaya H, Miyawaki O, Nakamura K (2001). Determination of log P for pressurized carbon dioxide and its characterization as a medium for enzyme reaction. Enzyme Microb Tech.

[33] Lou WY, Zong MH, Smith TJ (2006). Use of ionic liquids to improve whole-cell biocatalytic asymmetric reduction of acetyltrimethylsilane for efficient synthesis of enantiopure (*S*)-1-trimethylsilylethanol. Green Chem.

[34] Zhang BB, Cheng J, Lou WY, Wang P, Zong MH (2012). Efficient anti-Prelog enantioselective reduction of acetyltrimethylsilane to (*R*)-1-trimethylsilylethanol by immobilized Candida parapsilosis CCTCC M203011 cells in ionic liquid-based biphasic systems. Microb Cell Fact..

[35] Gao P, Li A, Lee HH, Wang DIC, Li Z (2014). Enhancing enantioselectivity and productivity of P450-catalyzed asymmetric sulfoxidation with an aqueous/ionic liquid biphasic system. Acs Catal.

[36] Pfruender H, Amidjojo M, Kragl U, Weuster-Botz D (2004). Efficient whole-cell biotransformation in a biphasic ionic liquid/water system. Angew Chem Int Edit.

[37] Gangu SA, Weatherley LR, Scurto AM (2009). Whole-Cell biocatalysis with ionic liquids. Curr Org Chem.

[38] Gorke J, Srienc F, Kazlauskas R (2010). Toward advanced ionic liquids. Polar, enzyme-friendly solvents for biocatalysis. Biotechnol Bioprocess Eng.

[39] Kohlmann C, Robertz N, Leuchs S, Dogan Z, Luetz S, Bitzer K, Na’amnieh S, Greiner L (2011). Ionic liquid facilitates biocatalytic conversion of hardly water soluble ketones. J Mol Catal B Enzym.

[40] De Winter K, Verlinden K, Kren V, Weignerova L, Soetaert W, Desmet T (2013). Ionic liquids as cosolvents for glycosylation by sucrose phosphorylase: balancing acceptor solubility and enzyme stability. Green Chem.

[41] Guo J-L, Mu X-Q, Xu Y (2010). Integration of newly isolated biocatalyst and resin-based in situ product removal technique for the asymmetric synthesis of (R)-methyl mandelate. Bioproc Biosyst Eng.

[42] Du PX, Wei P, Lou WY, Zong MH (2014). Biocatalytic anti-Prelog reduction of prochiral ketones with whole cells of Acetobacter pasteurianus GIM1.158. Microb Cell Fact.

[43] Chen WJ, Lou WY, Yu CY, Wu H, Zong MH, Smith TJ (2012). Use of hydrophilic ionic liquids in a two-phase system to improve Mung bean epoxide hydrolases-mediated asymmetric hydrolysis of styrene oxide. J Biotechnol.

[44] Wang ZY, Bi YH, Zong MH (2012). Regioselective enzymatic procedure for preparing 3′-*O*-stearoyl-6-azauridine by using Burkholderia cepacia lipase. Biotechnol Bioproc E.

[45] Xu P, Xu Y, Li XF, Zhao BY, Zong MH, Lou WY (2015). Enhancing asymmetric reduction of 3-chloropropiophenone with immobilized *Acetobacter* sp. CCTCC M209061 cells by using deep eutectic solvents as cosolvents. ACS Sustain Chem Eng..

[46] Chen XH, Lou WY, Zong MH, Smith TJ (2011). Optimization of culture conditions to produce high yields of active *Acetobacter* sp. CCTCC M209061 cells for anti-Prelog reduction of prochiral ketones. BMC Biotechnol..

[47] Cetinus SA, Oztop HN, Saraydin D (2007). Immobilization of catalase onto chitosan and cibacron blue F3GA attached chitosan beads. Enzyme Microb Tech.

[48] Chang MY, Juang RS (2005). Activities, stabilities, and reaction kinetics of three free and chitosan-clay composite immobilized enzymes. Enzyme Microb Tech.

[49] Li YN, Shi XA, Zong MH, Meng C, Dong YQ, Guo YH (2007). Asymmetric reduction of 2-octanone in water/organic solvent biphasic system with Baker’s yeast FD-12. Enzyme Microb Tech.

[50] Weuster-Botz D (2007). Process intensification of whole-cell biocatalysis with ionic liquids. Chem Rec.

